# A novel signature of combing cuproptosis- with ferroptosis-related genes for prediction of prognosis, immunologic therapy responses and drug sensitivity in hepatocellular carcinoma

**DOI:** 10.3389/fonc.2022.1000993

**Published:** 2022-09-27

**Authors:** Chuanbing Zhao, Zhengle Zhang, Tao Jing

**Affiliations:** Department of Pancreatic Surgery, Renmin Hospital of Wuhan University, Wuhan, China

**Keywords:** CRFs, cuproptosis, ferroptosis, drug sensitivity, immunotherapy

## Abstract

**Background:**

Our study aimed to construct a novel signature (CRFs) of combing cuproptosis-related genes with ferroptosis-related genes for the prediction of the prognosis, responses of immunological therapy, and drug sensitivity of hepatocellular carcinoma (HCC) patients.

**Methods:**

The RNA sequencing and corresponding clinical data of patients with HCC were downloaded from The Cancer Genome Atlas (TCGA), International Cancer Genome Consortium (ICGC), GSE76427, GSE144269, GSE140580, Cancer Cell Line Encyclopedia (CCLE), and IMvigor210 cohorts. CRFs was constructed using the least absolute shrinkage and selection operator (LASSO) algorithm. The analyses involved in the prognosis, response to immunologic therapy, efficacy of transcatheter arterial chemoembolization (TACE) therapy, and drug sensitivity were performed. Furthermore, the molecular function, somatic mutation, and stemness analyses were further performed between the low- and high-risk groups, respectively. In this study, the statistical analyses were performed by using the diverse packages of R 4.1.3 software and Cytoscape 3.8.0.

**Results:**

CRFs included seven genes (G6PD, NRAS, RRM2, SQSTM1, SRXN1, TXNRD1, and ZFP69B). Multivariate Cox regression analyses demonstrated that CRFs were an independent risk factor for prognosis. In addition, these patients in the high-risk group presented with worse prognoses and a significant state of immunosuppression. Moreover, patients in the high-risk group might achieve greater outcomes after receiving immunologic therapy, while patients in the low-risk group are sensitive to TACE. Furthermore, we discovered that patients in the high-risk group may benefit from the administration of sunitinib. In addition, enhanced mRANsi and tumor mutation burden (TMB) yielded in the high-risk group. Additionally, the functions enriched in the low-risk group differed from those in the other group.

**Conclusion:**

In summary, CRFs may be regarded not only as a novel biomarker of worse prognosis, but also as an excellent predictor of immunotherapy response, efficacy of TACE and drug sensitivity in HCC, which is worthy of clinical promotion.

## Introduction

Hepatocellular carcinoma (HCC) is one of the leading causes of cancer-related death worldwide (1). Over the years, a great body of novel therapies, such as immunologic therapy and targeted therapy, have prolonged the survival time of a portion of HCC patients (2–5). However, due to the delayed emergence, drug resistance, and heterogeneity of HCC, these treatments still do not achieve satisfactory outcomes for HCC patients (6–8). Accordingly, exploring a novel signature that can be served as not only a therapeutic target but also a novel biomarker of drug sensitivity and immunologic therapy for HCC may be one of the principal focuses for scholars in this field.

In recent years, cuproptosis and ferroptosis have been defined, respectively, which are distinctly different from other recognized regulated modes of cell death, including necroptosis and autophagy (9, 10). Additionally, it is reported that the sensitivity of HCC cells to targeted therapeutic agents may also be influenced by the regulative mechanism of ferroptosis (11, 12). More importantly, the therapeutic approaches to regulate copper homeostasis have been demonstrating encouraging anticancer results (13, 14). Thus, cuproptosis- and ferroptosis-related regulatory mechanisms are expected to be novel targets for HCC treatment. Cuproptosis-related genes (CRGs) and ferroptosis-related genes (FRGs) have been reported to predict the prognosis as well as the immune profile of HCC patients. However, whether the CRGs combined with FRGs could be applied as a predictor of prognosis, the immunotherapy response, and drug sensitivity in HCC have not been addressed.

In this study, we have constructed and validated CRFs for predicting the prognosis. In addition, we have explored the role of CRFs in immune characteristics, the efficacy of TACE and drug sensitivity, potential molecular function, and somatic mutation. Our results indicated that CRFs showed excellent predictive performance for prognosis and played a favorable role in assessing the responses of immunotherapy, efficacy of TACE, and drug sensitivity for patients with HCC in different subgroups.

## Materials and methods

### Data acquisition and preprocessing

**Figure 1:**
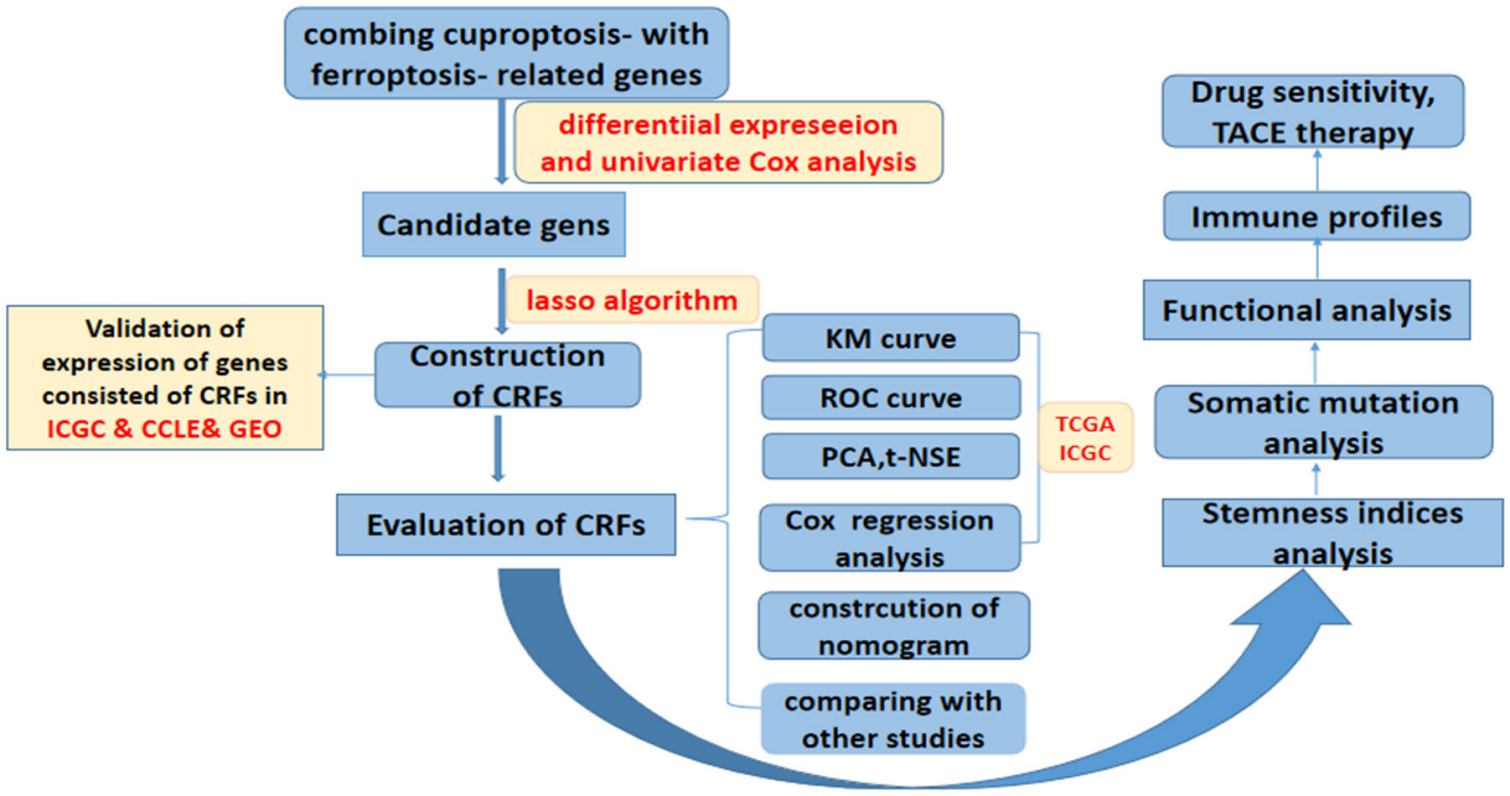
Flowchart of overall study design.

The RNA sequencing and corresponding clinical data of HCC patients were extracted from TCGA and ICGC databases. In addition, a list of CRGs and FRGs were derived from the existing publications (9) and FerrDb website (http://www.zhounan.org/ferrdb/) (15), respectively. In addition, the mRNA expression matrix and matching clinical data in GSE104580, GSE144269, and GSE76427 were extracted from Gene Expression Omnibus (GEO). The data of the expression and clinical information of patients in the IMvigor210 cohort were downloaded using “IMvigor210CoreBiologies” package (16). The data of the somatic mutation of LIHC were downloaded from the Genomic Data Commons (GDC) database, and the mRNA expression matrix on the HCC cell line was obtained from the CCLE database (https://portals.broadinstitute.org/ccle) (17). The expression matrix files downloaded from the GEO data set were normalized using “limma” and “sva” packages. The flowchart of this study is shown in **Figure 1**.

### Prognostic analysis

#### Construction and validation of cuproptosis- with ferroptosis-related gene signature

**Figure 2:**
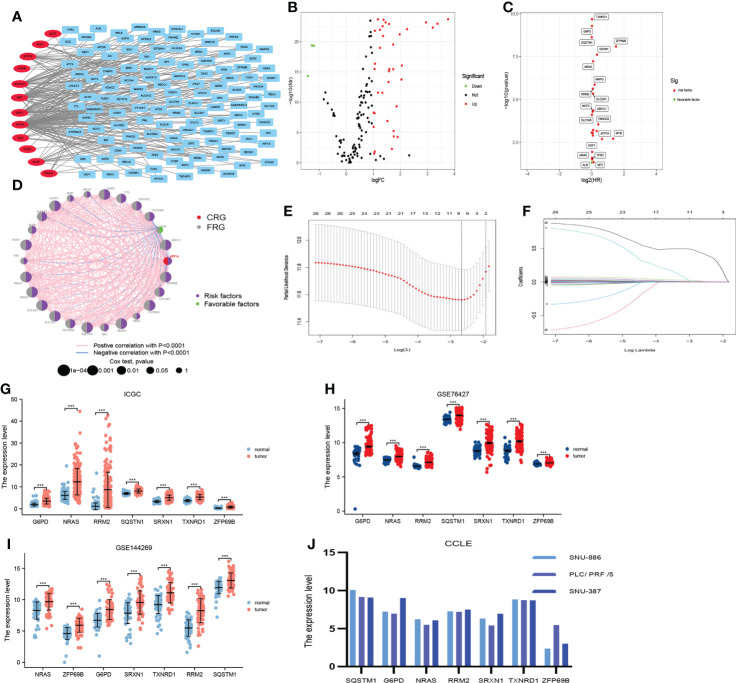
Construction of the prognostic signature (CRFs) in TCGA. **(A)** The correlation between cuproptosis-related genes and ferroptosis-related genes. **(B)** Differential expression of cuproptosis-related genes and cuproptosis–ferroptosis-related genes in hepatocellular carcinoma **(HCC)** versus normal tissues. **(C)** Univariate regression analysis of differentially expressed genes (DEGs). **(D)** The network of candidate genes. **(E)** Least absolute shrinkage and selection operator (LASSO) coefficient profiles. **(F)** Candidate genes were filtered by the LASSO algorithm. **(G)** Verification of the expression of genes constituting CRFs on patients with HCC in ICGC. **(H)** Verification of the expression of genes constituting CRFs on patients with HCC in GSE76427. **(I)** Verification of the expression of genes constituting CRFs on patients with HCC in GSE144269. **(J)** Verification of the expression of genes constituting CRFs on patients with HCC in CCLE.

First, we performed the correlation analysis of CRGs with FRGs in order to obtain particular FRGs (pFRGs) highly correlated with CRGs (|r|0.3, p<0.05). Additionally, we analyzed the differential expression of CRGs and pFRGs in HCC tissues versus normal liver tissues (|logFc|>1, FDR<0.05). In addition, univariate Cox regression analysis was employed to obtain candidate genes that were associated with a prognosis (|HR|>1.0, P<0.05). Moreover, the least absolute shrinkage and selection operator (LASSO) algorithm was applied to select seven genes constituting CRFs from candidate genes. The seven genes constituting CRFs were also highly expressed in HCC tissues based on data from the ICGC, GSE76427, GSE144269, and CCLE databases (**Figures 2F, G**). The risk scores were defined by multiplying the expression level of a gene by the coefficient of a variable, as follows:

Risk score =(the expression level of gene A *coefficient of gene A)+(the expression of gene B *coefficient of gene B)+……

Patients were classified into low- and high-risk groups according to the median risk score. In addition, the predictive performance of CRFs was evaluated by the receiver operating characteristic (ROC) curve, timeROC curve, Kaplan–Meier (KM) method, principal component analysis (PCA) and t-distributed stochastic neighbor embedding (t-NSE) *via* “survival”, “survminer”, “Rtsne”, “ggplot2”, “timeROC,” and “dplyr” packages. Additionally, the univariate and multivariate Cox regression analyses of risk scores and other clinical indicators were performed using the “survival” package. Furthermore, the risk score was calculated for all patients in the ICGC cohort and GSE144269 by the same formula as that used for the TCGA cohort and the predictive performances of CRFs for prognosis were validated in ICGC and GSE144269 cohort.

#### Comparison of cuproptosis- with ferroptosis-related gene signature with other gene signatures

To verify the predictive performance of CRFs for prognosis, we have further compared CRFs with other studies using the “ggDCA” and “rms” packages to identify whether CRFs were more valuable in predicting the overall survival (OS) of patients with HCC.

### The role of cuproptosis- with ferroptosis-related gene signature in predicting responses of immunological and other therapy

#### The role of cuproptosis- with ferroptosis-related gene signature for prediction of responses to immunotherapy

Single-sample gene set enrichment analysis (ssGSEA) was used to quantify the immune cells as well as the immune function of each sample with “GSVA” and “GSEABase” packages. In addition, the xCELL and CIBERSORT algorithms were applied to determine the abundance of immune cell infiltration in the tumor microenvironment in patients with HCC using the “immunedeconv” package. Furthermore, we identified if there were any differences in immune cells and immune functions between low- and high-risk groups. In addition, we further evaluated whether the expression of common immune checkpoints was varied in the high- and low-risk groups as well.

The “tumor immune dysfunction and exclusion” (TIDE) algorithm was being employed to calculate the TIDE score for each sample, which may be able to predict the response of immunologic therapy (18). Afterwards, we identified whether TIDE scores differed between the high- and low-risk groups. Additionally, we applied the TCIA database to verify whether there were any differences in response to PD-1 or CTLA-4 treatment between the high- and low- risk groups of patients with HCC (19).

It has been suggested that the T-cell-inflamed (TIS) score can be used to assess the efficacy of combined immunotherapy (20). Accordingly, we calculated the TIS score for each sample with the use of the “limma” package and further determined if there were any differences in TIS scores between the patients in these subgroups. A recent study has demonstrated that the responders to immune checkpoint inhibitors (ICIs) upregulated the expression of CD8A and STAT1 (21).

In addition, we calculated risk scores for each sample in the IMvigor210 cohort and analyzed the implications of risk scores on the efficacy of PD-L1 inhibitors with the “ limma” package.

#### Cuproptosis- with ferroptosis-related gene signature for prediction of responses of transcatheter arterial chemoembolization

TACE therapy is considered as an alternative for patients with unresectable HCC. Thus, it is clinically important to study the impact of risk scores on the efficacy of TACE. We calculated risk scores for each sample in the GSE104580 and analyzed the impact of risk scores on the responses of TACE using the “limma” package.

#### The role of cuproptosis- with ferroptosis-related gene signature for prediction of drug sensitivity

The “pRRophetic” package was employed to select potentially effective drugs from over 300 drugs for patients in high- and low-risk groups, respectively. Notably, we further performed correlation analysis between the drug sensitivity and risk score, with sensitivity indicators expressed as IC50 values.

### Other analyses

#### Stemness analysis

The one-class logistic regression (OCLR) algorithm was created by Malta, which was mainly applied to calculate mRNAsi (22). According to this algorithm, we calculated the mRNAsi for each sample. Furthermore, we further identified if there existed differences in mRNAsi between these subgroups.

#### Somatic mutation analysis

Given that genetic mutations may as well have implications for the prognosis of patients with HCC, we further determined whether there were any differences in genetic mutations between these subgroups. We initially analyzed the top 20 mutated genes in the high- and low-risk groups and visualized the mutation details of these genes as waterfall plots using the “maftool” package. In addition, we calculated the tumor mutation burden (TMB) for each sample and further made a comparison of TMB in these subgroups. Of note, the impact of TMB on the prognosis of HCC was studied as well using the “survival” package.

#### Functional analysis

Differentially expressed genes (DEGs) in the high- and low-risk groups were screened applying the”limma” package, and Gene Ontology (GO) and Kyoto Encyclopedia of Genes and Genomes (KEGG) analyses were performed on the DEGs using the “clusterProfiler” package. To determine the potential distinctions in the molecular mechanism and biological functions between these subgroups, we further performed the gene set enrichment analysis (GSEA) between high- and low-risk groups based on reference gene sets (symbols.gmt v7.4.) in the MSigDB database (https://www.gsea-msigdb.org/gsea/msigdb) with the use of “clusterProfiler,” “enrichplot,” and “circlize” packages.

### Statistical analyses

OS, disease-specific survival (DSS), and progression-free interval (PFI) were compared in different subgroups by using the KM method. We further performed the correlation analyses of risk scores and clinical indicators using the “ComplexHeatmap” package. Moreover, a nomogram was constructed and validated by the “rms” package. In this study, the statistical analyses were performed by using R 4.1.3 software and Cytoscape 3.8.0.

## Result

### The predictive performance of combining cuproptosis- with ferroptosis-related gene signature for prognosis

#### Construction of combining cuproptosis- with ferroptosis-related gene signature

A total of 122 FAGs were found to be strongly correlated with 12 CRGs (**Figure 2A**). In addition, we screened 40 differentially expressed genes among these 134 genes (**Figure 2B**). In addition, we obtained 26 candidate genes from these 40 genes by univariate Cox regression analysis (**Figures 2C, D**). Furthermore, these candidate genes were entered into the LASSO algorithm, and seven genes (G6PD, NRAS, RRM2, SQSTM1, SRXN1, TXNRD1, and ZFP69B) were selected for the construction of CRFs (**Figures 2E, F**). It was mentioning that the seven genes that comprised CRFs were highly expressed in HCC tissues according to the data from ICGC, GSE76427, GSE144269, and CCLE databases (**Figures 2G–J**).

CRFs risk score = [expression level of G6PD×(0.005568)] + [expression level of NRAS×(0.006045)] + [expression level of RRM2×(0.003086) + expression level of SQSTM1×(0.000544)] + [expression level of SRXN1×(0.015443)] + [expression level of TXNRD1×(0.003416)] + [expression level of ZFP69B×(0.436101)]

**Figure 3:**
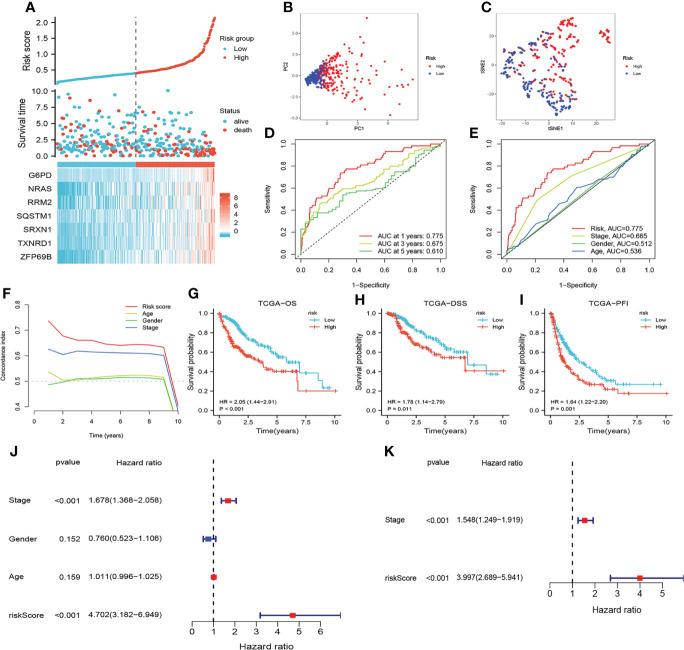
Assessment of the prognostic signature (CRFs) in TCGA. **(A)** survival status distribution. **(B)** Principal component analysis (PCA) plot. **(C)** t-Distributed stochastic neighbor embedding (t-NSE) plot. **(D)** timeROC curve of the risk score. **(E)** Receiver operating characteristic (ROC) curve of the age, gender, stage and riskScore. **(F)** C-index curve of the age, gender, stage and risk scores. **(G)** Kaplan–Meier (KM) curves of overall survival (OS). **(H)** KM curves of disease-specific survival (DSS). **(I)** KM curves of progression-free interval. **(J)** Univariate Cox regression analysis of the age, gender, stage, and risk score in TCGA. **(K)** Multivariate Cox regression analysis of stage and risk scores in TCGA.

The risk scores of HCC patients in the TCGA cohort were calculated according to this formula, and HCC patients were grouped into high-risk and low-risk groups based on the median risk scores. We identified that patients with higher risk scores showed shorter OS and higher mortality (**Figure 3A**). PCA and t-NSE indicated a significant clustering of HCC patients in the low- and high-risk groups (**Figures 3B, C**). As shown in **Figures 3D, E**, CRFs was a promising predictor of OS in patients with HCC. Additionally, KM curves showed that patients in the high-risk group presented a worse prognosis (P<0.01) (**Figures 3G–I**). Notably, this study demonstrated that CRFs was superior to the TNM stage in predicting the prognosis of patients with HCC (**Figure 3F**). Univariate Cox regression analysis indicated that CRFs was the risk factor for the OS of HCC patients [HR: 4.702, 95% confidence interval (CI): 3.182 to 6.949, p<0.001] (**Figure 3J**), and the multivariate Cox regression analysis showed that CRFs was an independent risk factor for the OS of HCC patients (in TCGA, HR: 3.997, 95% CI: 2.689-5.941, p<0.001) (**Figure 3K**).

#### Validation of combining cuproptosis- with ferroptosis-related gene signature

**Figure 4:**
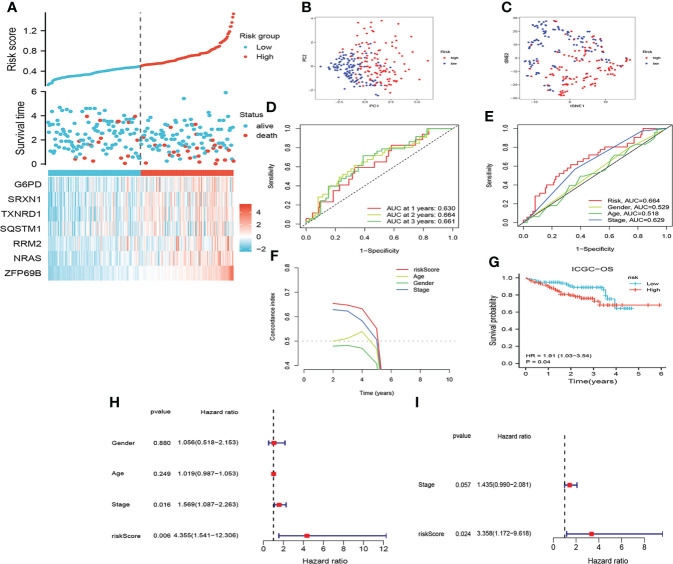
Assessment of the prognostic signature (CRFs) in ICGC. **(A)** Survival status distribution. **(B)** PCA plot. **(C)** t-NSE plot. **(D)** timeROC curve of the risk score.**(E)** ROC curve of the age, gender, stage, and risk scores. **(F)** C-index curve of the age, gender, stage, and risk scores. **(G)** KM curves of OS. **(H)** Univariate Cox regression analysis of the age, gender, stage, and risk score in ICGC. **(I)** Multivariate Cox regression analysis of the stage and risk scores in ICGC.

The risk score of all patients in the ICGC cohort were calculated by the same formula as that used for TCGA. Similarly, patients in the ICGC cohort were assigned to low- and high-risk group based on the median risk scores. As shown in **Figures 4A–I**, the results of assessing the prognosis of HCC by CRFs in the ICGC cohort were almost in line with those of the TCGA cohort. Furthermore, CRFs presented with excellent performances as well for the prediction of prognoses on HCC patients in the GSE144269 cohort (**Supplementary Figures S1A–C**). Accordingly, these findings suggested that CRFs showed great potential in predicting the prognoses of HCC.

#### Correlation of combining cuproptosis- with ferroptosis-related gene signature with clinicopathological parameters

**Figure 5:**
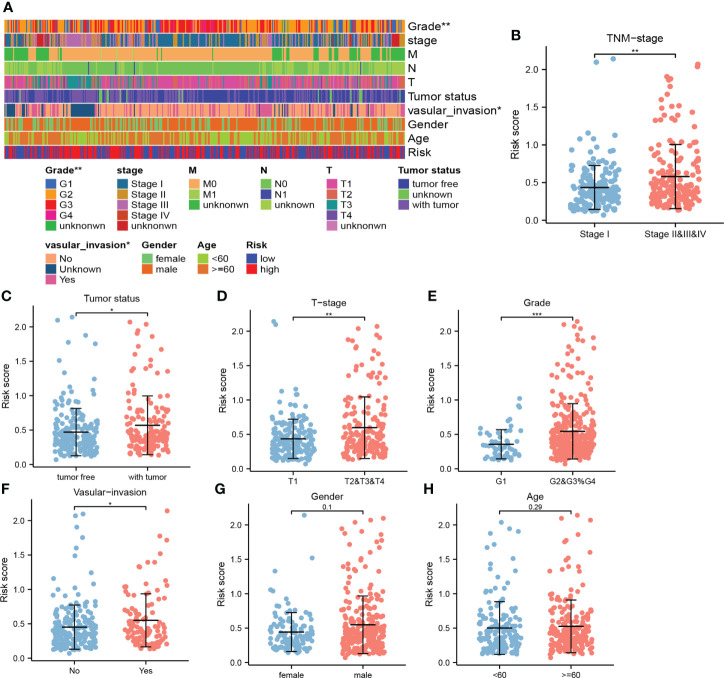
The correlation between the risk score and clinical indicators. **(A)** The correlation heat map. **(B)** Comparison of risk scores in stage I and stage II, III, and IV. **(C)** Comparison of risk scores in tumor- free and tumor status. **(D)** Comparison of risk scores in T1 and T2, 3, and 4. **(E)** Comparison of risk scores in G1 and G2, G3, and G4. **(F)** Comparison of risk scores in the presence and absence of vascular invasion. **(G)** Comparison of risk scores in men and women. **(H)** Comparison of risk scores at ages ≥60 vs ≤60 years old. (*, **, and *** represent p < 0.05, p < 0.01, and p < 0.001, respectively).

Higher risk scores were clustered in these patients with the advanced TNM stage, T stage, and grade (**Figures 5A, B, D, E, G, H**). In addition, relatively high risk scores were also observed in these patients with the tumor status and vascular invasion features (**Figures 5A, C, F**). This indicated that higher risk scores represented worse prognosis and verified the great potential of CRFs in predicting the prognosis of HCC.

#### Comparison of combining cuproptosis- with ferroptosis-related gene signature with other promising gene signatures

**Figure 6:**
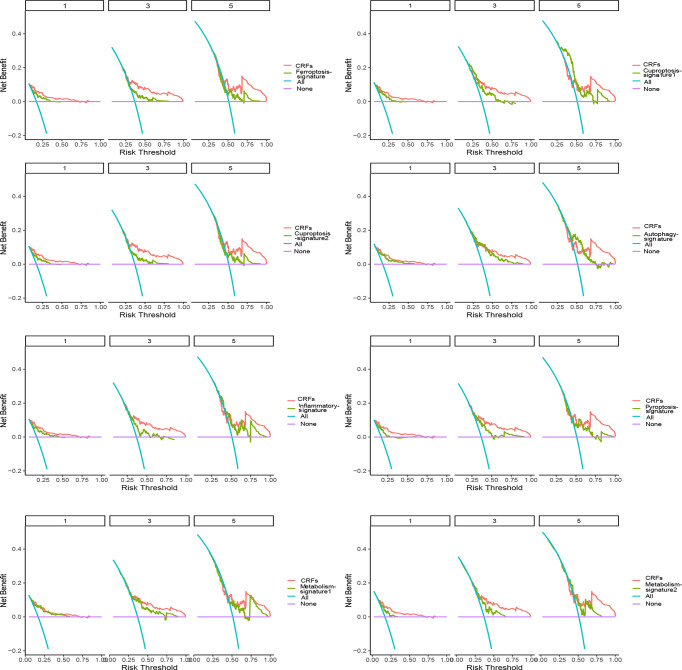
Comparison of CRFs with other gene signatures.

As shown in **Figure 6**, comparing with these studies involved in the cuproptosis-, pyroptosis-, inflammatory response-, ferroptosis-, and metabolism-related gene signatures (23–30), CRFs performed superiorly in predicting the prognosis of patients with HCC. Of great significance, our study further verified the strong predictive power of CRFs in assessing the OS of HCC.

#### Construction of a combining cuproptosis- with ferroptosis-related gene signature–based nomogram

**Figure 7:**
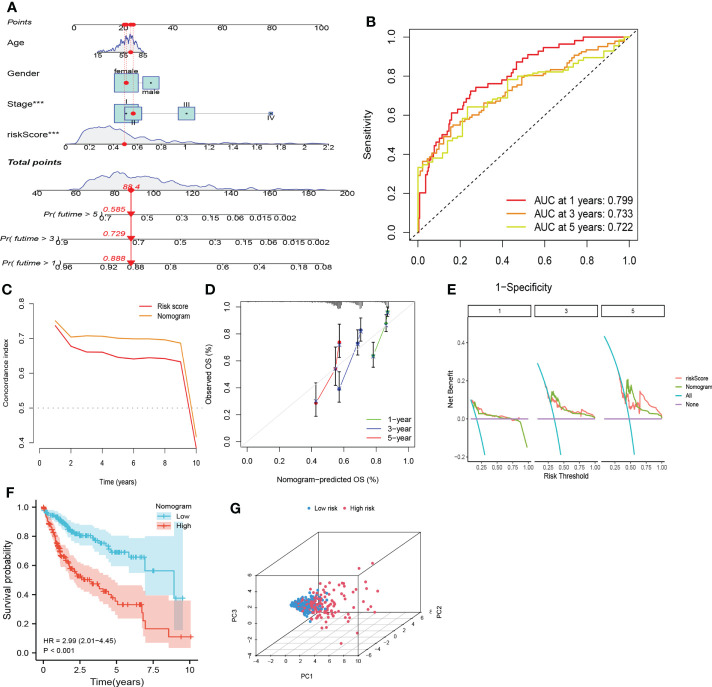
Nomogram based on CRFs, gender, and stage. **(A)** Nomogram. **(B)** timeROC curve of the nomogram. **(C)** C-index curve of the nomogram. **(D)**Calibration curve of the nomogram. **(E)** Decision curve analysis of the nomogram. **(F)** PCA curve of the nomogram. **(G)** KM curve of the nomogram.

To enable better application of CRFs in clinical practice, we created a nomogram based on the risk score, gender, TNM-stage, and age in the TCGA cohort (**Figure 7A**). As shown in **Figure 7B**, the AUC values for predicting 1-, 3-, and 5-year survival in HCC patients were 0.799, 0.733, and 0.722, respectively, while the c-index and DCA curves showed that the nomogram may be more accurate in predicting the prognosis of HCC patients compared with the risk score and TNM stage (**Figures 7C, E**). In addition, the calibration curves demonstrated that the predicted probabilities of 1-, 3-, and 5-year survival were highly consistent with the actual values (**Figure 7D**). Based on the median of the total scores of the nomogram, patients were stratified into high- and low-risk groups, respectively. Moreover, the nomogram could distinguish between high- and low-risk patients, and patients in the high-risk group showed worse OS (**Figures 7F, G**). These results suggested that the nomogram presented with excellent potential for predicting OS in HCC patients.

### The role of combining cuproptosis- with ferroptosis-related gene signature in predicting the responses to immunotherapy and other therapies

#### The correlation between combining cuproptosis- with ferroptosis-related gene signature and immune features

**Figure 8:**
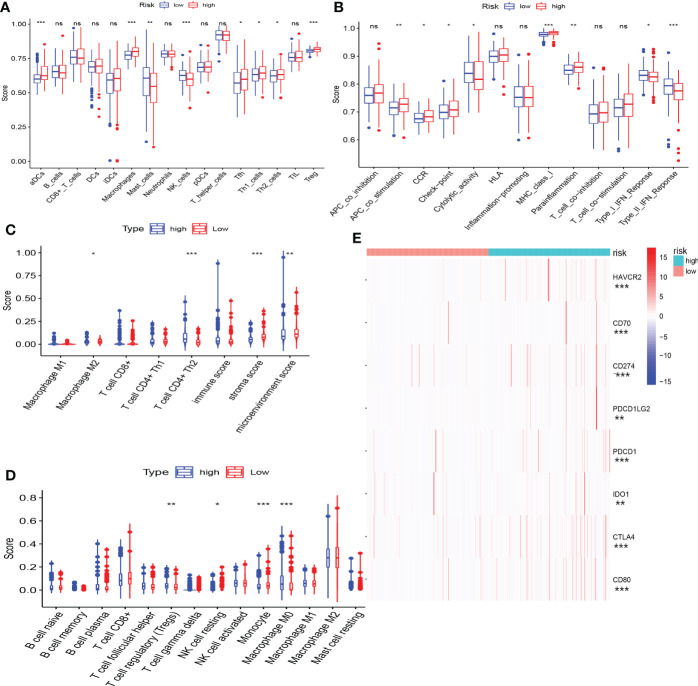
The correlation between CRFs and immune features. **(A)** Comparison of immune cell infiltration in high- and low-risk groups using single-sample gene set enrichment analysis. **(B)** Comparison of immune function in high- and low-risk groups using gene set enrichment analysis (GSEA). **(C)** Comparison of immune cell infiltration in high- and low-risk groups using xCELL. **(D)** Comparison of immune cell infiltration in high- and low-risk groups using CIBERSORT. **(E)** Comparison of the expression level of common immune checkpoints in high- and low-risk groups. (*, **, ***, and ns represent p < 0.05, p < 0.01, p < 0.001, and “not statistically” ,respectively.

The immune status of the high-risk and low-risk groups was significantly different. Patients in the low-risk group exhibited a greater proportion of macrophages and NK cells, whereas the high-risk group presented more Th2 and Treg cells (**Figures 8A, C, D**). In terms of immune function, type-I IFN response and type-II IFN response were significantly enhanced in the low-risk group, whereas immune checkpoints and parainflammation were upregulated in the high-risk group (**Figure 8B**). We discovered that patients in the high-risk group appeared to have a significant state of immunosuppression.

The expression of immune checkpoint genes was actually upregulated in the high-risk group (**Figure 8E**), and the risk scores were markedly positively related with the expression of some promising immune checkpoint genes, such as CTLA4, PDCD1, and CD274 (**Supplementary Figures S2 A-C**).

#### Combining cuproptosis- with ferroptosis-related gene signature for prediction of the responses to immunologic therapy

**Figure 9:**
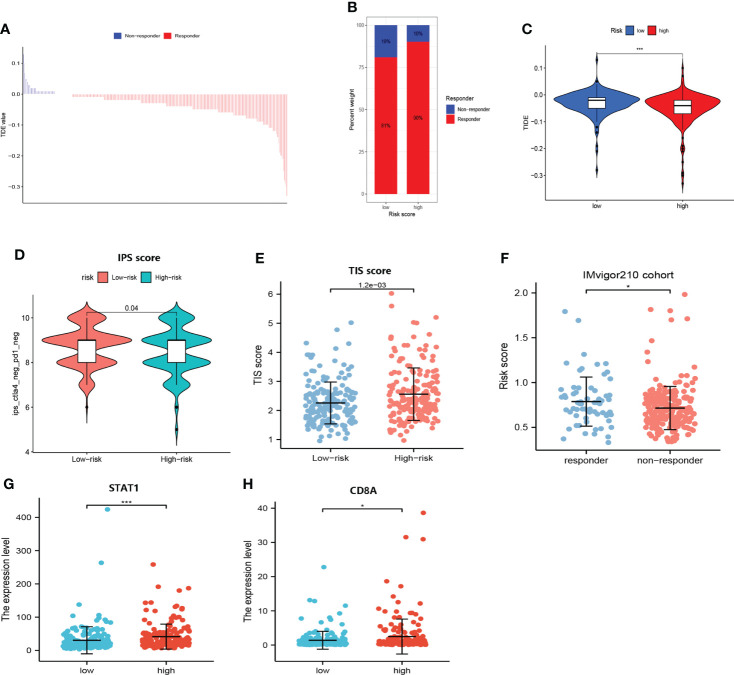
Responses of immunologic therapy. **(A)** The distribution of the TIDE score in responders and non-responders. **(B)** The proportion of responders and non-responders in the high- and low- risk groups, respectively. **(C)** Comparison of the TIDE score in high- and low-risk groups. **(D)** Comparison of the IPS score in high- and low-risk groups. **(E)** Comparison of the TIS score in high- and low-risk groups. **(F)** Comparison of risk scores in responders and non-responders to immunotherapy in the IMvigor210 cohort. **(G)** Comparison of the expression level of STAT1 in high- and low-risk groups. **(H)** Comparison of the expression level of CD8A in high- and low-risk groups. (* and *** represent p < 0.05 and p < 0.001, respectively.).

As shown in **Figure 9A**, the responders of immunological therapy presented with lower TIDE scores. Of great importance, patients in the high-risk group may be more likely to respond to immunological therapy (**Figures 9B, C**). Additionally, the immunophenoscore scores identified that patients in the low-risk group were not sensitive to the treatment of PD-1 and CTLA-4 (**Figure 9D**). Furthermore, patients in the high-risk group showed higher TIS scores (P<0.05) (**Figure 9E**). In addition, the upregulated expression of CD8A and STAT1 was clustered in the high-risk group (**Figures 9G, H**). As expected, elevated risk scores yielded in these responders to immunotherapy in the IMvigor210 cohort (**Figure 9F**). Our results suggested that patients in the high-risk group may be more likely to benefit from immunologic therapy.

#### Combining cuproptosis- with ferroptosis-related gene signature for prediction of responses to transcatheter arterial chemoembolization

**Figure 10:**
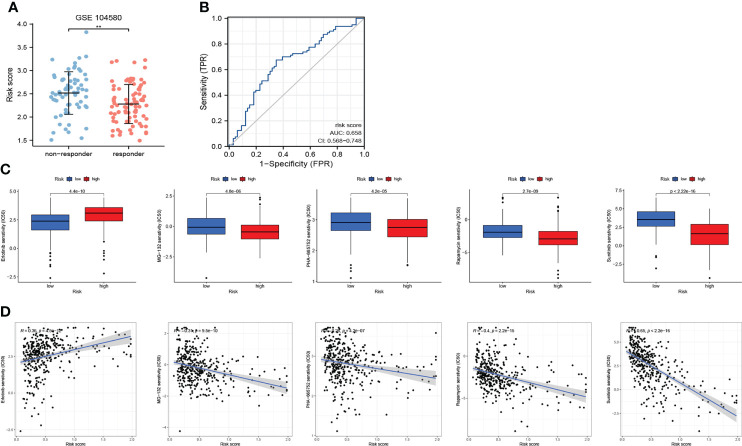
The role of CRFs in drug sensitivity and responses to transcatheter arterial chemoembolization (TACE). **(A)** Comparison of the risk score in responders and non-responders to TACE in GSE140580. **(B)** ROC curve of the risk score in determining the responses to TACE. **(C)** Comparison of drug sensitivity in high- and low-risk groups. **(D)** The correlation of risk scores and drug sensitivity.

As shown in **Figure 10A**, patients who responded to TACE treatment presented with lower risk scores (p<0.01). In addition, the AUC for the risk score assessing the TACE response was 0.658 (AUC value >0.65) (**Figure 10B**). These results initially demonstrated that CRFs may be applied as a novel biomarker to evaluate the efficacy of TACE therapy in patients with HCC.

#### Combining cuproptosis- with ferroptosis-related gene signature for prediction of drug sensitivity

We selected five drugs from over a total of 300 drugs, of which MG-132, PHA-665752, rapamycin, and sunitinib were more suitable for patients in the high-risk group, while erlotinib may better benefit patients in the low-risk group (P<0.001) (**Figure 10C**). More importantly, we also obtained correlation coefficients between risk scores and drug sensitivity (**Figure 10D**), from which we demonstrated that sunitinib may be most suitable to patients in the high-risk group.

### Other analyses

#### mRNAsi analyses

**Figure 11:**
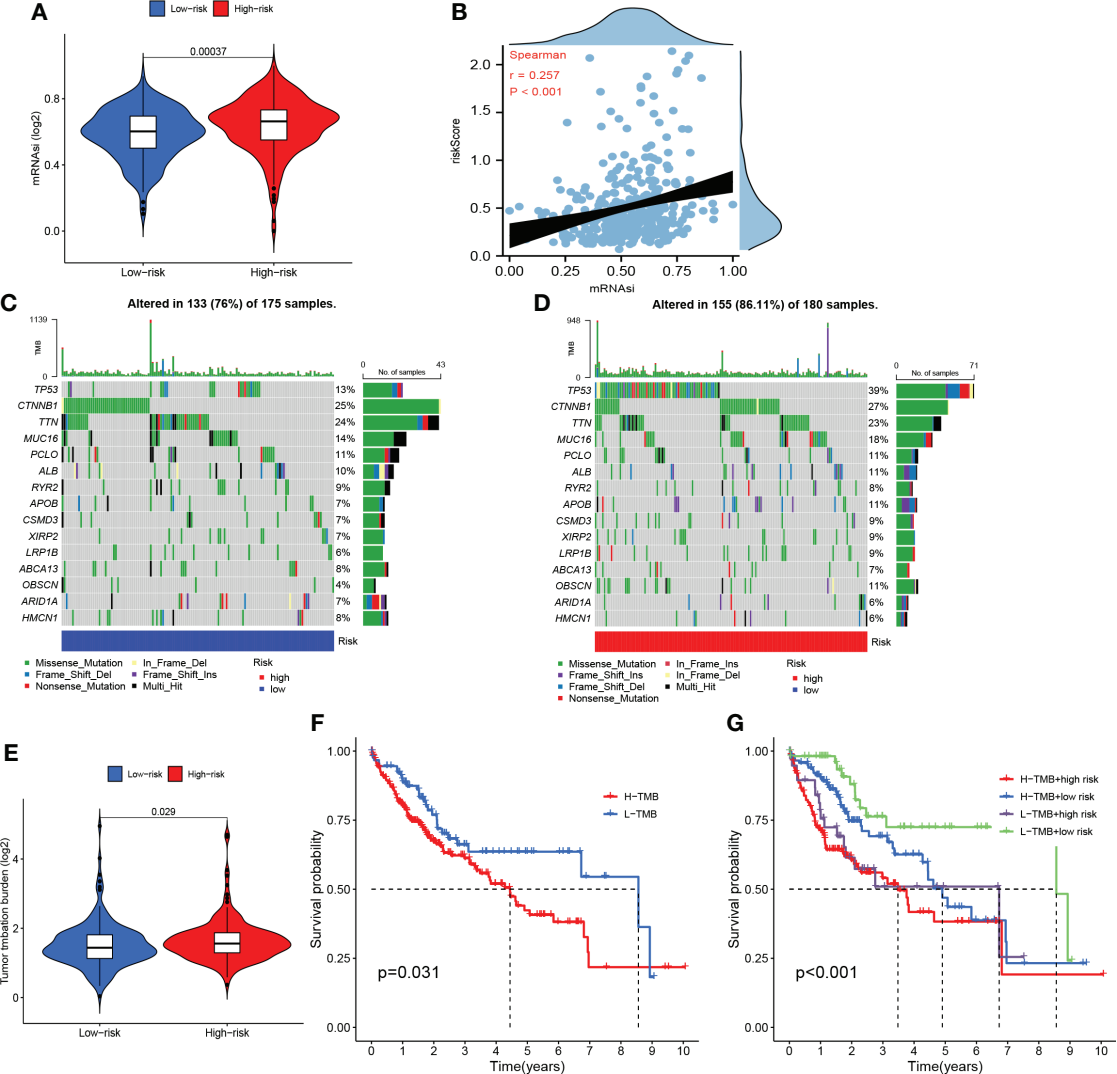
Somatic mutation and stemness analysis. **(A)** Comparison of mRNAsi in high- and low-risk groups. **(B)** The correlation between the risk score and mRNAsi. **(C)** The top 20 mutated genes in the high-risk group. **(D)** The top 20 mutated genes in the low-risk group. **(E)** Comparison of tumor mutation burden (TMB) in high- and low-risk groups. **(F)** KM curve of TMB. **(G)** KM curve of TMB + CRFs.

As shown in **Figures 11A, B**, patients in the high-risk group presented with a higher proportion of mRNAsi and the risk scores were positively related to mRNAsi. These findings may interpret one of major mechanisms of the poor prognoses of patients in the high-risk group from the perspective of mRNAsi.

#### Somatic mutation analyses

According to the waterfall plot, we discovered that TP53 mutation was more frequent in the high-risk group while CTNNB1 mutation was more common in the low-risk group (**Figures 11C, D**). Patients in the high-risk group presented with higher frequency of mutations than those in the low-risk group. In addition, missense mutations were the most common type of mutation in both subgroups. As shown in **Figure 11E**, the TMB in the high-risk group was significantly greater than that in the low-risk group. Moreover, the OS of patients within the high-TMB group was inferior to that of low-TMB patients (**Figure 11F**). It is worth mentioning that TMB may perform better in predicting OS when TMB was combined with the risk score (**Figure 11G**).

#### Molecular function analyses

**Figure 12:**
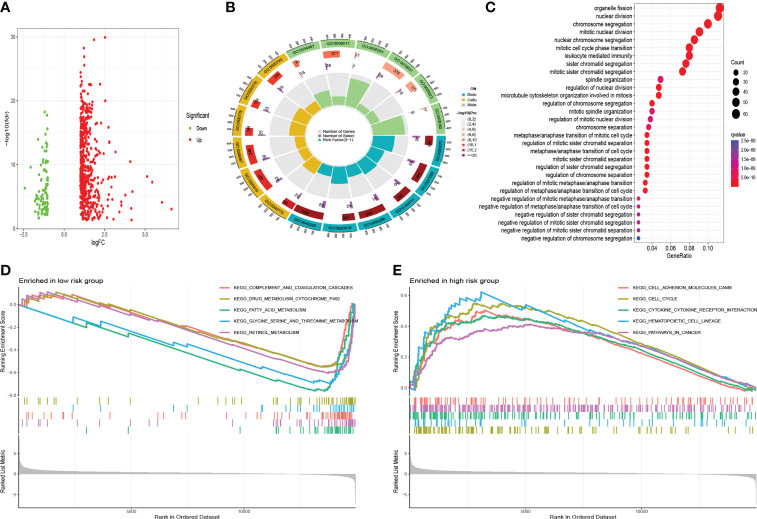
Functional analysis. **(A)** The volcano of DEGs. **(B)** Gene Ontology analysis. **(C)** KEGG analysis. **(D)** GSEA in the low-risk group. **(E)** GSEA in the high-risk group.

The DEGs between high- and low- risk groups were visualized by a volcano plot (**Figure 12A**). As it is shown in **Figures 12B, C** and **Table 1**, the functions of these genes were mostly enriched in organelle fission, nuclear division, and chromosome segregation. GSEA revealed that many cancer metastatic pathways were significantly enriched in the high-risk group, such as cell adhesion molecules (CAMs), cytokine–receptor interactions, the cell cycle, and pathways in cancer (**Figure 12E**). Interestingly, the hematopoietic cell lineage–related pathway was also significantly enriched in the high-risk group. In addition, some metabolic pathways were enriched in the low-risk group, such as fatty acid, glycine serine, threonine metabolism, and drug metabolism P450-related pathways (**Figure 12D**).

**Table 1:** The description of Gene Ontology terms in **Figure 12B**.

ONTOLOGY	ID	Description	GeneRatio	p.adjust
CC	GO:0000775	Chromosome, centromeric region	32/565	9.47E-13
CC	GO:0005819	Spindle	42/565	5.07E-11
CC	GO:0098687	Chromosomal region	39/565	5.35E-11
CC	GO:0000779	Condensed chromosome, centromeric region	25/565	5.35E-11
CC	GO:0000776	Kinetochore	23/565	4.71E-10
BP	GO:0000070	Mitotic sister chromatid segregation	40/550	1.34E-21
BP	GO:0007059	Chromosome segregation	55/550	1.34E-21
BP	GO:0140014	Mitotic nuclear division	50/550	1.95E-21
BP	GO:0000280	Nuclear division	61/550	2.48E-21
BP	GO:0000819	Sister chromatid segregation	42/550	5.42E-21
CC	GO:0000775	Chromosome, centromeric region	32/565	9.47E-13
CC	GO:0005819	spindle	42/565	5.07E-11
CC	GO:0098687	Chromosomal region	39/565	5.35E-11
CC	GO:0000779	Condensed chromosome, Centromeric region	25/565	5.35E-11
CC	GO:0000776	Kinetochore	23/565	4.71E-10

## Discussion

In this study, CRFs was created by using the data from the TCGA cohort and validated in the ICGC and GSE144269 cohorts. Our results revealed that CRFs presented with excellent performances in predicting the prognosis of HCC patients. Moreover, mRNAsi, the landscape of genetic mutations, and molecular functions in both groups of patients were distinctly different. Additionally, marked differences were observed in the TME and immune cell infiltration between these subgroups. Furthermore, CRFs may be able to predict the sensitivity of immunologic therapy and TACE. Of great importance, we screened effective drugs for HCC patients in the high- and low-risk groups, respectively. These findings strongly suggested great potential of CRFs in the prognosis and treatment of HCC.

Both cuproptosis and ferroptosis have been reported to be involved in the progression of a number of malignant tumors. However, the combination of CRGs and pFAGs have not been studied in HCC. Accordingly, examining the role of CRGs and pFAGs in HCC may have significant implications for the prognosis, immune profile, and drug sensitivity of HCC patients. We screened seven genes consisting of CRFs from 122 pFAGs and 12 CRGs, including G6PD, NRAS, RRM2, SQSTM1, SRXN1, TXNRD1, and ZFP69B. Several studies have revealed that G6PD may facilitate the proliferation and metastasis of HCC cells, as well as inhibit the ferroptosis of HCC cells (31, 32). In addition, NRAS may inhibit the sensitivity of HCC cells through the RAS/Raf/MEK/ERK signaling pathway, thus promoting HCC progression (33). Wang et al. discovered that paclitaxel can inhibit RRM2, which is one of the mechanisms by which paclitaxel inhibits the proliferation of HCC cells (34). Moreover, it has been suggested that SQSTM1 may serve as a prognostic biomarker for HCC (35). And the overexpression of SRXN1 has been reported to stimulate the migration and invasion of HCC cells (36). Targeting TXNRD1 leads to a dramatic accumulation of ROS, which is deemed to be an effective way to suppress HCC tumor growth (37). ZFP69B has been reported to predict the prognosis of gastric cancer (38). Nevertheless, the role of ZFP69B in HCC has not been previously studied, and this study may lay the foundation for future studies on ZFP69B in HCC.

CRFs could serve as a novel biomarker to predict the prognosis of HCC, as demonstrated in TCGA, ICGC, and GSE144269. More specifically, CRFs was able to distinguish between the high- and low- risk groups of HCC patients. In addition, patients in the high- risk group were more likely to show shorter survival time and a higher mortality rate. Moreover, CRFs have been shown to be an independent risk factor for the poor prognosis of HCC. Notably, the nomogram based on the risk score and TNM stage demonstrated excellent accuracy and discriminatory power in predicting the prognosis of HCC. We discovered that the predictive performance of the nomogram was superior to that of the risk score and TNM-stage, and the nomogram may be more suitable for clinical application. Our results indicated the excellent performance of CRFs in assessing the prognosis of HCC patients.

What exactly resulted in the significant differences in prognosis between the high- and low-risk groups of patients with HCC?

Could it be the markedly different genetic mutations in the high- and low-risk groups that contributed to this outcome? Our results demonstrated that patients in the high-risk group showed a higher frequency of genetic mutations. It has been reported that higher genomic instability was closely related with worse OS, compared to lower genomic instability (39). In addition, TP53 and CTNNB1 mutations were most common in the patients of high- and low-risk groups, respectively. Recent evidence suggests that HCC tissues with CTNNB1 mutation are characteristic of better differentiation and a lower grade (40). Nevertheless, HCC tissues with TP53 mutation are characterized by hypodifferentiation, vascular invasion, and angiogenesis (40). As a result, the landscape of genetic mutations may be helpful to account for one of the possible reasons for the markedly different prognosis of patients in high- and low-risk groups.

The distinct molecular mechanism may be the other reason leading to this outcome. The molecular functions of the high risk were enriched in cancer metastatic pathways, including CAMs, the cell cycle, cytokine–receptor interaction, hematopoietic cell lineage, and pathways in cancer. On the other hand, the functions in the low risk were enriched in metabolism-related pathways, such as drug metabolism cytochrome-p450, fatty acid metabolism, glycine sering and threonine metabolism and retinol metabolism. Taken together, the diverse molecular pathways may be the underlying mechanism for the varying prognosis of high- and low-risk groups.

Furthermore, our results revealed that patients in the high-risk group presented with higher mRNAsi. Additionally, higher mRNAi has been reported to be typically positively correlated with the dedifferentiation and aggressiveness of tumor cells (41). This may also account for the poorer prognosis of patients in the high-risk group.

Immune characteristics may be another factor contributing to the above phenomenon. ssGESA analysis revealed that the infiltration levels of Th2 and Treg cells were observed in the high-risk group, while NK cells were more abundant in the low-risk group. There is growing evidence that Th2 cells and Treg cells may promote the occurrence of the immune escape of malignant tumors, including HCC (42). In addition, NK cells exert a powerful antitumor immune response (43). There existed remarkable differences as well in the immune function between the high- and low-risk groups. Moreover, the expression of immune checkpoints was mainly pronounced in the high-risk group, whereas the function of type-I IFN and type-II IFN responses appeared to be stronger in the low-risk group. It was worth noting that the immunosuppression status of the patients may contribute to the poorer prognosis of patients in the high-risk group.

CRFs has demonstrated a significant clinical value in predicting the response to immunotherapy, the efficacy of TACE, and drug sensitivity in HCC patients. Thus, CRFs may play a favorable role in guiding personalized treatment for patients with HCC.

Over the years, immunologic therapy has been a promising alternative for a large number of HCC patients. Nevertheless, it is not all patients who are sensitive to immunotherapy (ICIs). In addition, there is an urgent need to distinguish from responders and non-responders to immunotherapy. A wealth of studies demonstrated that the efficiency of immunologic therapy always depends on the adequate expression of immune checkpoints in the tumor tissues (44, 45). In this study, the expression level of common checkpoints upregulated in the high-risk group indicating that patients in the high-risk group were more likely to gain benefits from immunotherapy. In addition, the TIDE, TIS, and IPS scores reaffirmed that patients in the high-risk group may be more likely to achieve satisfactory outcomes from immunologic therapy. Furthermore, the enhanced TMB yielded in the patients of high-risk group and emerging evidence demonstrated that higher TMB indicated better clinical outcomes after receiving immunologic therapy (46). It has been reported that the overexpression of CD8A and STAT1 is more likely to be observed in ICI responders, while our results showed elevated CD8A and STAT1 expression in the high-risk group. It is worth mentioning that immunotherapy responders showed greater risk scores compared to non-responders in the IMvigor210 cohort (P<0.05). More importantly, CTNNB1 and TP53 mutations were more common in the low- and high-risk group, respectively. Additionally, such HCC patients with higher CTNNB1 mutation are less likely to respond to immunologic therapy (47). TP53 mutation has been proven to be applied as a promising biomarker for the prediction of response to PD-1 immunotherapy (48). Taken together, CRFs could provide a meaningful reference to immune interventions for patients with HCC from various perspectives.

Notably, our results indicated that lower risk scores were clustered in responders to TACE treatment (P<0.01) in the GSE104580 cohort. In addition, the AUC value exceeded 0.65, suggesting that CRFs may be used to assess the response to TACE in patients with HCC. However, the mechanisms underlying this phenomenon still need to be further explored.

Chemotherapy and targeted therapy play vital roles in the treatment of advanced HCC patients. According to CRFs, effective drugs for patients in high- and low-risk groups were selected, respectively. Notably, sunitinib may be more effective for patients in the high- risk group based on CRFs.

Our study is the first to explore the role of CRGs combined with pFRGs in HCC, with relatively profound implications. Of significance, compared with other promising gene signatures, CRFs demonstrated superior performances in evaluating the prognosis of patients with HCC. Notably, this study indicated that patients in the high-risk group might be sensitive to immunologic therapy from diverse perspectives. Of course, there existed some limitations in this study. Similar to many publications (49–52), we only extracted data from public databases and did not validate these data with animal experiments and clinical specimens. In addition, the nomogram lacked imaging data, which are also significant for assessing the prognosis of HCC.

In summary, our study defined a novel gene signature based on seven cuproptosis-related ferroptosis genes and demonstrated that CRFs performed excellently in predicting the prognosis of patients with HCC. Of note, patients in the high-risk group were more likely to respond to immunologic therapy, while patients in the low-risk group may benefit from TACE. Based on CRFs, sunitinib was proven to be more suitable to patients in the high-risk group. Therefore, CRFs may be expected to be applied as a novel biomarker for prognosis, immunologic therapy, TACE therapy, and drug sensitivity.

## Disclosure

TCGA, ICGC and GEO belong to public databases. The patients involved in the database have obtained ethical approval. Users can download relevant data for free for research and publish relevant articles. Our study is based on open-source data, so there are no ethical issues and other conflicts of interest.

## Data availability statement

The original contributions presented in the study are included in the article/**Supplementary Material**. Further inquiries can be directed to the corresponding author.

## Author contributions

CZ: Data analysis, Writing- Original draft preparation. ZZ: Writing- Original draft preparation. TJ: Conception and design, Final approval of the version to be published.

## Conflict of interest

The authors declare that the research was conducted in the absence of any commercial or financial relationships that could be construed as a potential conflict of interest.

## Publisher’s note

All claims expressed in this article are solely those of the authors and do not necessarily represent those of their affiliated organizations, or those of the publisher, the editors and the reviewers. Any product that may be evaluated in this article, or claim that may be made by its manufacturer, is not guaranteed or endorsed by the publisher.

## Supplementary material

Supplementary Figure 1 Assessment of the prognostic signature (CRFs) in GSE144269. **(A)** survival status distribution **(B)** PCA plot. **(C)** t-NSE plot. **(D)** timeROC curve of risk score. **(E)** KM curves of OS.

Supplementary Figure 2 The correlation between the expression level of promising immune checkpoints. **(A)** The correlation between risk scores and PCDD1. **(B)** The correlation between risk scores and CD274 (PD-L1). **(C)** The correlation between risk scores and CTLA-4.

## Abbreviations

HCC, hepatocellular carcinoma; LASSO, least absolute shrinkage and selection operator; TCGA, The Cancer Genome Atlas; TMB, tumor mutation burden; OS, overall survival; TME, tumor microenvironment; CRGs, cuproptosis-related genes; FRGs, ferroptosis-related genes; pFRGs, particular ferroptosis-related genes that are highly correlated with cuproptosis-related genes; CRFs, combining cuproptosis- with ferroptosis-related gene signature.
