# Supplementary figures and images for: A novel signature of combing cuproptosis- with ferroptosis-related genes for prediction of prognosis, immunologic therapy responses and drug sensitivity in hepatocellular carcinoma
Source: Front Oncol. 2022 Sep 27;12:1000993. doi: 10.3389/fonc.2022.1000993 (PMC9562991; doi:10.3389/fonc.2022.1000993)

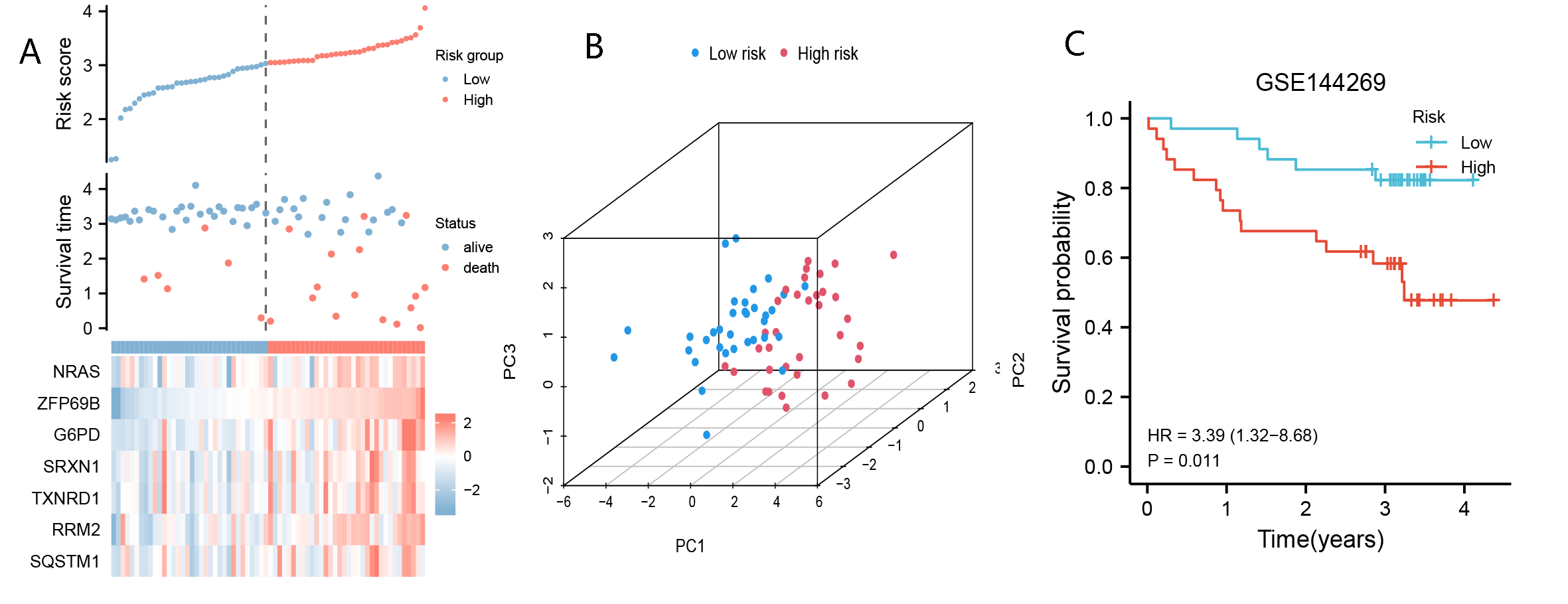

Supplement: Supplementary Figure 1 — Assessment of the prognostic signature (CRFs) in GSE144269. (A) survival status distribution (B) PCA plot. (C) t-NSE plot. (D) timeROC curve of risk score. (E) KM curves of OS. [file Image_1.tif]

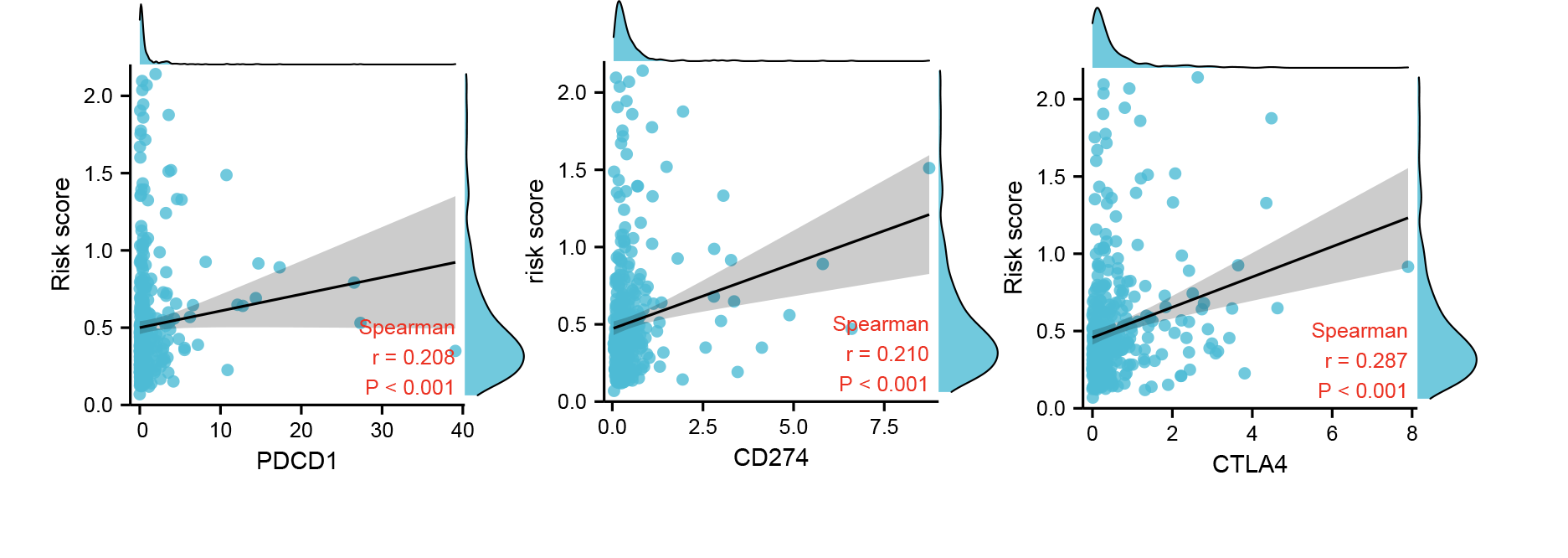

Supplement: Supplementary Figure 2 — The correlation between the expression level of promising immune checkpoints. (A) The correlation between risk scores and PCDD1. (B) The correlation between risk scores and CD274 (PD-L1). (C) The correlation between risk scores and CTLA-4. [file Image_2.tif]
